# Use of UPLC-ESI-MS/MS to quantitate free amino acid concentrations in micro-samples of mammalian milk

**DOI:** 10.1186/2193-1801-2-622

**Published:** 2013-11-20

**Authors:** Véronique Ferchaud Roucher, Emmanuelle Desnots, Charlotte Naël, Aurore Martin Agnoux, Marie-Cécile Alexandre-Gouabau, Dominique Darmaun, Clair-Yves Boquien

**Affiliations:** CRNH, Human Nutrition Research Center, Nantes, F-44093 France; LUNAM Université, Nantes, F-44 200 France; IMAD, DHU 2020, CHU Hôtel-Dieu, Nantes, F-44 000 France; INRA, UMR 1 280 Physiologie des Adaptations Nutritionnelles, Nantes, F-44 000 France

**Keywords:** Free amino acid, Human milk, Rat milk, Cow milk, UPLC-ESI-MS/MS

## Abstract

Although free amino acids (FAA) account for a small fraction of total nitrogen in mammalian milk, they are more abundant in human milk than in most formulas, and may serve as a readily available source of amino acids for protein synthesis, as well as fulfill specific physiologic roles. We used reversed phase Ultra Performance Liquid Chromatography (UPLC) coupled to electrospray ionization tandem mass spectrometry (ESI-MS/MS) technique for FAA profiling in milks from three species (human, rat and cow) with a simple and rapid sample preparation. The derivatization procedure chosen, combined with UPLC-ESI-MS/MS allowed the quantitation of 21 FAA using labeled amino acids (Internal Standards) over a 10 min run time in micro-samples of mammalian milk (50 μL). The low limit of quantitation was 0.05 pmol/μL for most FAA with good repeatability and reproducibility (mean CV of 5.1%). Higher levels of total FAA were found in human (3032 μM) and rat milk (3460 μM) than in bovine milk (240 μM), with wide differences in the abundances of specific FAA between species. This robust analytical method could be applied to monitor FAA profile in human breast milk, and open the way to individualized adjustment of FAA content for the nutritional management of infants.

## Introduction

Due to its many health benefits, breastfeeding is considered the gold standard for feeding infants in the first 6 months of life (Horta et al. 2007). The composition of milk therefore has long been the focus of intense scrutiny in humans as well as other mammalian species (Jensen 1995). Among macronutrients, protein is of key importance since milk protein content largely determines the initial growth rate of the pups. Protein concentration varies by a factor of 10 among mammals, with the lowest content for humans (10 to 11 g/L) (Jensen 1995), an intermediate content for cows (34 g/L) (Lindmark-Mansson et al. 2003) and the highest for rodents (80 to 100 g/L) (Grigor et al. 1986), and marine mammals (110 to 130 g/L) (Arnould and Boyd 1995). Across species, the higher the milk protein content, the higher the initial postnatal growth rate (Riek 2008), and the bound amino acid content of milk protein have long been documented both in humans (Yamawaki et al. 2005) and in other species (Lindmark-Mansson et al. 2003).

Besides protein, mammalian milk contains many non-protein nitrogen compounds including urea, ammonia, creatine, creatinine, amines, and free amino acids (FAA). Although, FAA account for only 2% of total nitrogen in human milk (Carratù et al. 2003), they may serve as a source of readily available nitrogen, particularly in the small intestine of preterm infants with limited proteolytic enzymatic activity (Schanler and Garza 1987), and thus impact early postnatal growth. In addition, besides their role as building blocks for protein synthesis, several amino acids have a specific physiologic role, and their availability during the first few weeks of life may be of crucial importance. For instance, adequate dietary taurine availability is required for a normal retinal structure in animals (Imaki et al. 1993), and taurine intake over the first months of life correlates with developmental quotient several years later (Wharton et al. 2004). Accordingly, most infant formulae have been supplemented with taurine to match the needs of babies. Other free amino acids may also be *conditionally essential* in the first few weeks of life: in a porcine model, maternal arginine supplementation was shown to increase sows’ milk arginine concentration, and enhance piglet growth (Mateo et al. 2008).

FAA have been already analyzed in human (Agostoni et al. 2000; Atkinson et al. 1980; Chuang et al. 2005; Pamblanco et al. 1989; Sarwar et al. 1998), cow, and other mammalian (Grigor et al. 1986; Sarwar et al. 1998; Wu and Knabe 1994) milks. Yet, to the best of our knowledge, the full FAA profile has not been reported in rats although the mother-reared rat pup model has been the most commonly animal model used in neonatal nutrition. Allowing the determination of the FAA concentrations in rat milk should help nutritionists in the understanding of physiological effects observed in breastfed pups.

Multiple analytical methods have been developed for amino acid quantitation in various biological matrices, including the combination of different techniques of chromatography and various detection systems such as ion exchange chromatography amino acid analyzer (Chuang et al. 2005, Yamawaki et al. 2005), capillary electrophoresis-fluorescence (Lin and Liu 2004), LC-UV, LC-fluorescence (Carratù et al. 2003), GC-MS (Namera et al. 2002, Kaspar et al. 2008), and LC-MS (Dietzen et al. 2008). Two approaches have been commonly used: direct analysis, and precolumn derivatization methods (Jochum et al. 2006). Although fast and simple, the direct analysis presented insufficient specificity and sensitivity due to the lack of sufficient separation of amino acid in complex biological samples. The use of precolumn derivatization methods improved the sensitivity of amino acid detection but considerably lengthened sample preparation time. Over the last decade, the AccQ.Tag technology was developed using ultra-high performance liquid chromatography (UPLC) coupled with mass spectrometry, and resulted in improved efficiency, sensitivity, and limit of detection, and shortened the time of analysis of free amino acids, compared with conventional ion exchange techniques (Armenta et al. 2010). Although this analytical method was applied in various biological matrices (Armenta et al. 2010; Salazar et al. 2012) it has never been applied to human milk or other mammalian milks.

The aim of this work was to develop, optimize and validate a micro-assay that would require minimal sample preparation and run time, and that would be suitable for minimal amounts of milk. We used reversed phase liquid chromatography coupled to electrospray ionization tandem mass spectrometry (ESI-MS/MS) technique for FAA profiling in milks from three species (human, rat and cow) with a simple and rapid sample preparation. The AccQ.Tag derivatization combined with UPLC-ESI-MS/MS offered gains in selectivity and sensitivity using multiple reaction monitoring (MRM) and allowed the quantitation of 21 FAA using labeled amino acid internal standards over a 10 min run time, and in a small volume of mammalian milk (50 μL) particularly adapted for rodents. This robust analytical method could be applied to monitor FAA profile in human breast milk, and open the way to individualized adjustment of FAA content for the nutritional management of infants.

## Materials and methods

### Chemicals and reagents

Amino acid standards were purchased from Waters (Milford, USA), and L-glutamic acid, L-citrulline, L-glutamine, L-tryptophan, taurine, and L-alanine from Sigma-Aldrich (Saint-Quentin Fallavier, France). L-[1-^13^C]alanine, L-[2-^15^ N]tyrosine, L-[2-^15^ N]glutamic acid, L-[2-^15^ N]citrulline and L-[^13^C_6_]arginine, to serve as internal standards were purchased from Cambridge Isotope Laboratories Inc (Andover, USA), L-[1-^13^C]arginine from Masstrace Inc (Woburn, USA), L-[2-^15^ N]aspartic acid, L-[2-^15^ N_3_]histidine_,_, L-[2-^15^ N]serine, L-[2-^15^ N]threonine, L-[^2^H_5_]tryptophan, [^15^ N]taurine, L-[2-^15^ N]glutamine, L-[1-^13^C]glycine, and L-[^2^H_5_]phenylalanine were purchased from Sigma, L-[2-^15^ N]leucine from Tracer Technologies, inc (Waterloo, Canada), and L-[1-^13^C]valine from Eurisotop (Saint-Aubin, France). Ultrapure water was obtained from a Milli-Q purifier (Millipore, Eschborn, Germany), UPLC-grade solvents, derivation reagents, and UPLC column were purchased from Waters (Milford, USA), acetonitrile was from Biosolve (Valkenswaard, Netherlands), hydrochloric acid (HCl) from CARLO ERBA (Val de Rueil, France), and sulfosalicylic acid from Sigma-Aldrich.

### Milk samples

Human milk was obtained from the biocollection that has been established at Nantes University Hospital (Number DC-2009-982). Samples were kept at -80°C from collection until analysis. Eighteen samples were obtained after one month of lactation. Cow’s milk was obtained from 4 cows from the same herd by milking them manually at the end of the lactation period (8 months). Rat milk was obtained from an animal study conducted in accordance with the European Communities Council Directive of November 24^th^ 1986 (86/609/EEC) regarding the care and use of animals for experimental procedures and were approved by the Institut National de la Recherche Agronomique (INRA, Paris, France). Animal facility was approved by the French Veterinary Department and was registered under the number A44276. The experimental protocol was registered under the number CEEA.2011.4. Rat milk was collected as described by Grigor *et al*. (Grigor et al. 1986) at the end of the lactation (18 days). Briefly, pups were removed from their dams for 2 hours before milk was collected. Dams were anesthetized with a mix of isofluran and oxygen, on mats heated during the manipulation, and received an intraperitoneal injection of oxytocin (1 unit of Syntocinon; Sigma-Tau, Ivry-sur-Seine, France) to stimulate milk let-down. After twenty minutes, dams were shaved and disinfected with ethanol, and milk was collected by applying manual pressure to all nipples, until milk flow ceased. Around 0.5 mL of milk was obtained from each animal, and was immediately frozen to -80°C.

### Sample preparation

Standard solutions were prepared for each amino acid in 0.1 M HCl, and stored at -20°C until analysis. A mixture was prepared from 21 natural unlabeled amino acids mixed at relevant concentrations (depending of FAA concentration in milk) to create an unlabeled standard pool solution, and diluted with 0.1 M HCl. A labeled standard pool solution was prepared with 17 labeled amino acids mixed under the same conditions and used as an internal standard. The different points of concentration calibration were prepared by adding increasing amounts of unlabeled standard pool to the labeled internal standard pool reaching concentration ranges from 2.5 to 50 μM for tryptophan, the less concentrated FAA in milk, and from 100 to 2000 μM for glutamic acid, the more concentrated FAA in milk.

### Free amino acids extraction

Because of the small sample volume obtained in small rodents, the FAA extraction was optimized for use on a 50 μL sample. Prior to extraction, frozen milk samples were thawed on ice. One hundred microliters of labeled internal standard pool were added into a 200 μL aliquot of each milk sample (human and cow milk). One hundred fifty microliters of ultrapure water and 25 μL of labeled internal standard pool were added to 50 μL of rat milk.

All samples were mixed and defatted by centrifugation at 10 000 g for 15 min at 4°C. The upper (lipid) phase was removed (200 μL), and proteins were precipitated in the aqueous supernatant by adding 20 μL of a 10% (wt/v) solution of sulfosalicylic acid (SSA) in water, followed by centrifugation at 10 000 g for 15 min at 4°C. The supernatant phase was collected.

### AccQ•Tag™ ultra derivatization

FAA were derivatized by adding a mixture of 70 μL of AccQ•Tag™ Ultra Borate Buffer (Armenta et al. 2010), 10 μL of supernatant and 20 μL of AccQ•Tag™ Ultra reagent (6-aminoquinolyl-N-hydroxysuccinimidyl carbamate), followed by incubation for 10 min at 55°C.

### LC-MS/MS parameters

#### UPLC analysis

Liquid chromatographic separation was performed on an Acquity H-Class® UPLC system (Waters Corporation, Milford, USA) equipped with a quaternary solvent manager, an autosampler maintained at 4°C, a Waters AccQ•TagTM Ultra column (2.1 mm × 10 mm, 1.7 μm particles) with a pre-filter heated at 55°C, and coupled with a tandem quadrupole detector. Initial tests were performed with the gradient recommended in the AccQ•Tag UltraTM assay kit for amino acids from Waters developed for UV detection, but the peak resolutions were not sufficient for MS detection. The nonlinear separation gradient used was reported by Armenta *et al*. (2010), and is detailed in Table 1 with a mobile phase flow rate of 0.7 mL/min. One microliter of sample was injected in duplicate into the UPLC system.

**Table 1:** **Gradient elution used for free amino acid analysis (Armenta et al.** 2010 **), Eluent A was 10% AccQ•Tag**™ **Ultra concentrate solvent A in deionized water and eluent B was 100% AccQ•Tag**™ **Ultra solvent B**

Chromatographic gradient program over UPLC-MS/MS analysis time (10 min)			
Time (min)	Mobile phase	Mobile phase	Flow rate
	A (%)	B (%)	(mL/min)
0	99.9	0.1	0.7
0.54	99.9	0.1	0.7
5.74	90.0	10.0	0.7
7.74	78.8	21.2	0.7
8.04	40.4	59.6	0.7
8.64	40.4	59.6	0.7
8.73	99.9	0.1	0.7
10.00	99.9	0.1	0.7

#### ESI-MS/MS analysis

UPLC-MS/MS analysis was carried out on a Xevo TQD® (Waters Corporation, Milford, U.S.A.). We used parameters of detection as detailed by Armenta et al. (2010). The ESI source was operated at 150°C with a desolvatation temperature of 450°C, a 900 L/h desolvatation gas flow rate and a capillary voltage set at 3.2 kV. The extractor voltage was set at 3.0 V, and the radio frequency voltage at 2.5 V. The cone voltage varied from 27-39 V, depending on the amino acid investigated. Argon was used as the collision gas, and collision energies varied from 19 to 35 eV. Multiple Reaction Monitoring (MRM) was performed in the positive mode. Integration and quantitation were performed using the Waters TargetLinksTM software.

#### Quantitative analysis

Calibration curves were constructed by plotting the peak area ratios of unlabeled standard and internal standard (IS) versus the concentration of each amino acid, and used to determine the concentrations of each FAA in milk samples.

### Method validation

The method was validated for linearity and limit of quantitation, recovery, within- and between-sample repeatability, and reproducibility. The upper and lower limits of detection were determined and defined the domain of linearity of the method. Recovery (expressed as percent of added amount) was determined by the analysis of aliquots of human milk spiked with known amounts of unlabeled FAA. The between-sample repeatability was established by analyzing 10 supernatants obtained by extraction from the same sample of human milk. The within-sample repeatability was determined by repeatedly injecting on the same day the same extract or the same calibration solution into the instrument. Reproducibility was evaluated by having the same milk sample extracted by two different operators, and analyzed 6 times on different days.

## Results

### Method performance

The method was optimized and validated for quantification of 21 FAA in every milk tested.

### FAA extraction optimization

Preliminary experiments were performed to optimize extraction yield and chromatographic profile. Protein precipitation was tested with methanol and SSA (Agostoni et al. 2000; Chuang et al. 2005) with graded volumes of SSA, 20 μL, 100 μL, and 200 μL, and at two different SSA concentrations (10% and 35%). The best peak profiles were observed with 20 μL of 10% SSA. Milk delipidation was also tested and FAA recovery was similar after milk delipidation. We therefore elected to include the delipidation and deproteinization steps in the procedure in order to inject a sample as pure as possible into the ESI-MS/MS instrument.

### Derivatization optimization

In preliminary experiments graded volumes of AccQ•Tag™ Ultra reagent were tested to optimize derivatization. As the 6-aminoquinolyl-N-hydroxysuccinimidyl carbamate (AQC) was in large excess, we tested a lower volume of reagent. Only 10 μL of AccQ•Tag™ Ultra reagent in 80 μL of borate buffer and 10 μL of standard or supernatant milk sample were added, and a comparison was made between three ranges conducted under both conditions (data not shown). The slopes were similar, as were the coefficients of determination. Our ability to detect peaks of aminoquinolyl (AMQ) and di-urea aminoquinolyl (di-AMQ) (high intensity) ensured that reagents were consistently in excess. For all subsequent derivatization, the protocol therefore used 10 μL of AccQ•Tag™ Ultra reagent and 80 μL of borate buffer for human and cow milk or 60 μL for rat milk, with resp. 10 μL of supernatant (human/cow milk) or 30 μL of supernatant (rat milk).

### ESI- MS/MS optimization

**Figure 1:**
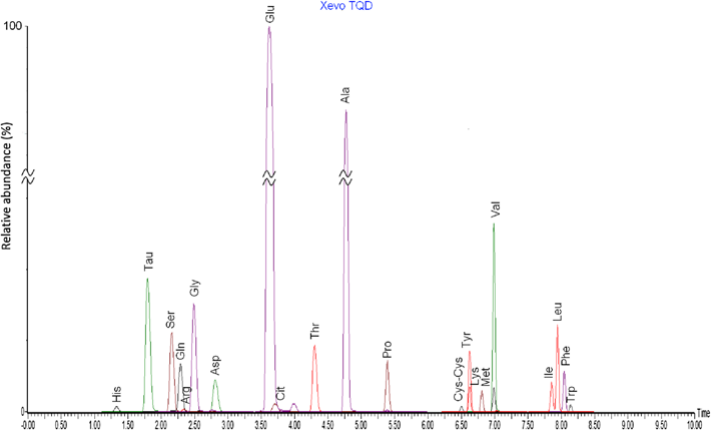
**Ion chromatogram obtained for FAA in human milk (cumulative).**

To optimize the MS/MS parameters and create a MRM method, each derivatized amino acid was first injected by direct infusion into the tandem quadrupole mass spectrometer. In our case, the drift (AQC) largely in excess of the product derivation (AMQ) was found highly concentrated compared to the amounts of amino acids present in sample suggested a strong contamination of the mass analyzer after direct infusion. Transitions of the majority of amino acids are reported by Armenta *et al*. (2010). For other amino acids like glutamine, lysine and cystine, the default setting was used (Table 2), by injecting 25 μM solutions on column. The predominant ions and retention time were detected by MS Scan. Each amino acid produced the same daughter ion (m/z = 171), corresponding to the cleavage of ureide bond of the AccQ•Tag adduct in each amino acid derivative. The run time was divided into five segments distributed over the duration of the liquid chromatographic run (Table 2). Figure 1 presents the ion chromatogram obtained from a representative sample of human milk. The dwell time for each amino acid was optimized to obtain sufficient data points for accurate quantitation. The MRM transitions, cone voltages, collision energies, and corresponding internal standards are listed in Table 2.

**Table 2:** **ESI-MS/MS conditions for quantitation of FAA in milk**

Compound number	Amino acid/ labeled amino acid	MRM transition (m/z)	Cone voltage (V)	Collision energy (eV)	Rt (min)	Function	Window time (min)*	Internal standard
1	L-Histidine	326.21 >171	35	19	1.33	1	1.1 – 2.0	2
2	L-[2-^15^ N_3_]Histidine	329.21 > 171	35	19	1.33	1	1.1 – 2.0	
3	Taurine	296.11 > 171	32	24	1.76	1	1.1 – 2.0	4
4	[^15^ N]Taurine	297.11 > 171	32	24	1.76	1	1.1 – 2.0	
5	L-Serine	276.11 > 171	27	19	2.12	2	1.9 – 3.4	6
6	L-[2-^15^ N]Serine	277.11 > 171	27	19	2.12	2	1.9 – 3.4	
7	L-Glutamine	317.14 > 171	35	20	2.26	2	1.9 – 3.4	8
8	L-[2-^15^ N]Glutamine	318.14 > 171	35	20	2.26	2	1.9 – 3.4	
9	L-Arginine	345.21 > 171	35	19	2.34	2	1.9 – 3.4	10
10	L-[1-^13^C]Arginine	347.21 > 171	35	19	2.34	2	1.9 – 3.4	
11	Glycine	246.16 > 171	33	20	2.47	2	1.9– 3.4	12
12	L-[1-^13^C]Glycine	247.16 > 171	33	20	2.47	2	1.9 – 3.4	
13	L-Aspartic acid	304.11 > 171	32	24	2.78	2	1.9 – 3.4	14
14	L-[2-^15^ N]Aspartic Acid	305.11 > 171	32	24	2.78	2	1.9 – 3.4	
15	L-Glutamic acid	318.11 > 171	32	24	3.62	3	3.4 – 6.0	16
16	L-[2-^15^ N]Glutamic Acid	319.11 > 171	32	24	3.62	3	3.4 – 6.0	
17	L-Citrulline	346.21 > 171	32	24	3.72	3	3.4 – 6.0	18
18	L-[2-^15^ N]Citrulline	347.21 > 171	32	24	3.72	3	3.4 – 6.0	
19	L-Threonine	291.11 > 171	31	22	4.30	3	3.4 – 6.0	20
20	L-[2-^15^ N]Thréonine	291.11 > 171	31	22	4.30	3	3.4 – 6.0	
21	L-Alanine	260.00 > 171	32	25	4.76	3	3.4 – 6.0	22
22	L-[1-^13^C]Alanine	261.00 > 171	32	25	4.76	3	3.4 – 6.0	
23	L-Proline	286.16 > 171	29	21	5.37	3	3.4 – 6.0	22
24	L-Cystine	291.18 > 171	32	24	6.50	4	6.2 – 7.5	31
25	L-Tyrosine	352.21 > 171	32	24	6.61	4	6.2 – 7.5	26
26	L-[2-^15^ N]Tyrosine	353.21 > 171	32	24	6.61	4	6.2 – 7.5	
27	L-Lysine	487.11 > 171	32	24	6.63	4	6.2 – 7.5	28
28	L-[^13^C_6_]Lysine	493.23 > 171	32	24	6.63	4	6.2 – 7.5	
29	L-Methionine	320.21 > 171	32	24	6.80	4	6.2 – 7.5	31
30	L-Valine	288.23 > 171	35	24	6.98	4	6.2 – 7.5	31
31	L-[1-^13^C]Valine	289.23 > 171	35	24	6.98	4	6.2 – 7.5	
32	L-Isoleucine	302.20 > 171	39	35	7.85	5	7.5 – 8.5	34
33	L-Leucine	302.20 > 171	39	35	7.94	5	7.5 – 8.5	34
34	L-[2-^15^ N]Leucine	303.20 > 171	39	35	7.94	5	7.5 – 8.5	
35	L-[2H5]Phenylalanine	341.21 > 171	32	24	8.01	5	7.5 – 8.5	
36	L-Phenylalanine	336.21 > 171	32	24	8.04	5	7.5 – 8.5	35
37	L-[^2^H_5_]Tryptophan	380.23 > 171	32	24	8.10	5	7.5 – 8.5	
38	L-Tryptophan	375.23 > 171	32	24	8.13	5	7.5 – 8.5	37

*Note: Before 1.10 min and after 8.50 min the flow state was in waste.

### Method validation

**Figure 2:**
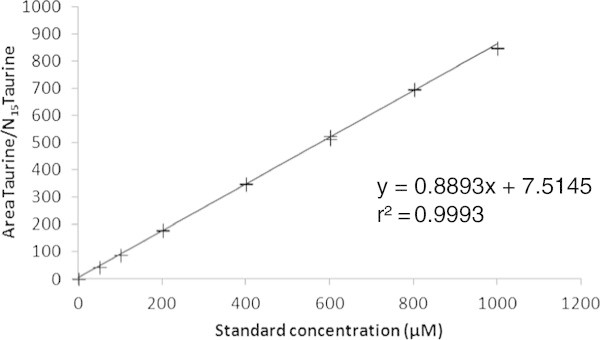
**Internal calibration curve for taurine.**

A pool of standard solutions containing 21 amino acids and 17 internal standards was prepared and diluted with 0.1 N HCl to appropriate concentrations. Two replicates of a diluted solution aliquot were injected into the instrument to determine the limits of quantitation and the dynamic range of the method. Results are presented in Table 3, and an example of calibration curve obtained for taurine is given in Figure 2. The R^2^ value of the regression analysis over the linear range exceeded 0.99 for all FAA. The LLOQ was 0.05 pmol/μL for most FAA corresponding to a limit of detection (LOD) of 15 fmol/μL.

**Table 3:** **Calibration data, dynamic range and human milk reproducibility of FAA (n = 6)**

Free amino acid	Linear regression data	Dynamic range	CV (%)^b^
	r^2^	pmol/μL^a^	
L-Histidine	0.9998	0.10 – 133.3	2.8
Taurine	0.9993	0.05 – 100	1.8
L-Serine	0.9994	0.05 – 26.7	2.3
L-Glutamine	0.9982	0.05 – 100	8.3
L-Arginine	0.9956	0.1 – 6.7	10.0
Glycine	0.9991	0.05 – 26.7	2.3
L-Aspartic acid	0.9980	0.05 – 26.7	2.9
L-Glutamic acid	0.9986	0.05 – 200	1.6
L-Citrulline	0.9974	0.05 – 5	8.6
L-Threonine	0.9990	0.05 – 26.7	2.8
L-Alanine	0.9953	0.05 – 53.3	2.2
L-Proline	0.9984	0.2 – 53.3	7.1
L-Cystine	0.9948	0.05 – 53.3	15.5
L-Tyrosine	0.9958	0.05 – 26.7	2.7
L-Lysine	0.9954	0.1 – 6.7	8.5
L-Methionine	0.9973	0.05 – 26.7	2.7
L-Valine	0.9973	0.05 – 26.7	2.5
L-Isoleucine	0.9947	0.05 – 13.3	4.4
L-Leucine	0.9968	0.05 – 13.3	2.9
L-Phenylalanine	0.9982	0.05 – 26.7	7.0
L-Tryptophan	0.9986	0.05 – 5	7.5

^a^assay concentration of AQC amino acid derivatives, 1 μL injected.

^b^coefficient variation of reproducibility, calculated on 6 independent milk samples tested on 2 days with 2 operators.

For all amino acids tested, the recoveries calculated by adding known amounts of natural amino acids to aliquots of human milk, exceeded 95%. In milk, regarding within-sample repeatability, the mean coefficients of variation (CV%) obtained were 1.6%, and ranged between 0.2% (for taurine) and 4.2% (for arginine). As for the between-sample repeatability, the mean CV was 2.2% and ranged between 0.6% (for glutamic acid) and 4.5% (for arginine), except for lysine and cystine (5.6%). Regarding reproducibility, the mean CV was 5.1%. All CV obtained for reproducibility are presented in Table 3.

### Application to human and mammalian milk analysis

**Figure 3:**
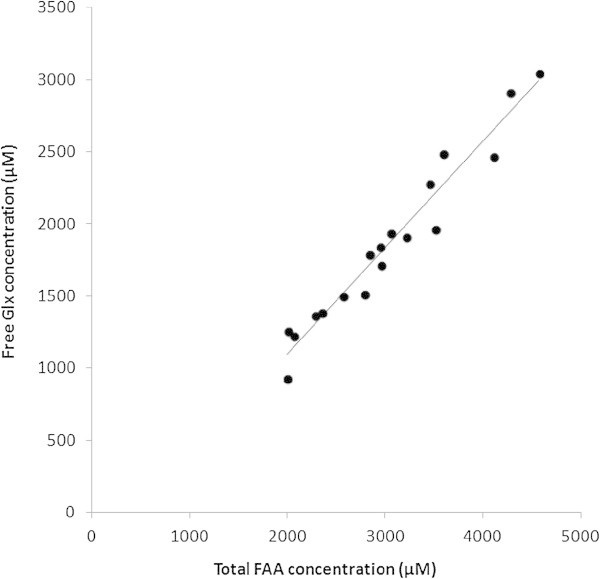
**Correlation between the total glutamic acid and glutamine (Glx) concentration and total FAA concentration in human milk samples (R** ^**2**^ **= 0.95).**

The LLOQ calculated in human milk samples were within 0.75-3 μM taking into account the dilution factor corresponding to a LOD within 0.2-0.9 μM. Mean concentrations for 21 FAA for human, bovine and rat mature milks are reported in Table 4. Higher levels of total FAA were found in human milk (3032 μM), and rat milk (3460 μM), compared with bovine milk (240 μM). Because of the conversion of glutamine to glutamic acid, the sum of both concentrations was calculated and is annotated as Glx in the text. Glx was by far the most abundant FAA (1861 μM) of human milk, accounting for about 61% of the total FAA, followed by taurine (311 μM) (10%), and alanine (226 μM) (7.5%). For all analyzed samples of breast milk, the sum of glutamine and glutamic acid correlates well (R^2^ = 0.97) with the sum of FAA (Figure 3). Glx was the most abundant FAA in bovine milk as well (136 μM), accounting for about 56% of the total FAA, followed by several amino acids at very similar levels representing only 5-8% of total FAAs (lysine (20 μM), alanine (15 μM), glycine and proline (13 μM)).The most abundant FAA in rat milk clearly was alanine (679 μM) (20%), followed by proline (424 μM) (12%), and Glx (413.5 μM) (12%). The essential amino acids (EAA) and the amino acids considered conditionally essential for human newborns (Arg, Ile, Leu, Lys, Met, Phe, Thr, Trp, Val) had low concentrations in breast milk, compared with non essential amino acids (EAA/NEAA of 10%), whereas essential amino acids represented a higher proportion of total amino acids in rat milk (with an EAA/NEAA ratio of 42%). Sulfur amino acids (Tau, cys-cys, Met) represented between 3.9% and 11.7% of total amount of FAA in the milk of the three species tested.

**Table 4:** **FAA in human milk (at 1 month of lactation), cow milk, and rat milk (Values are reported as means, standard deviation, minimum and maximum)**

Free amino acids μmol/L	Human milk (n = 16)				Rat milk (n = 8)				Cow milk (n = 4)			
	Mean	SD	Min	Max	Mean	SD	Min	Max	Mean	SD	Min	Max
Alanine	225.9	73.4	123.1	385.1	679.0	239.8	261.8	990.9	11.8	10.7	0.8	26.2
Arginine	19.5	12.4	1.5	42.8	53.8	34.0	14.1	119.1	10.7	4.9	3.8	15.4
Aspartic Acid	68.8	36.1	23.2	146.7	225.7	47.7	171.0	320.1	5.4	5.5	0.8	12.3
Citrulline	7.1	3.2	1.9	13.6	42.5	21.4	9.3	68.7	<LLOQ	-	-	-
Cystine	21.0	8.7	11.2	43.0	33.1	21.3	4.2	64.2	<LLOQ	-	-	-
Glx	1,860.7	586.2	926.0	3,040.4	413.5	102.2	212.4	528.0	136.2	64.5	58.9	216.5
Glycine	112.7	37.3	61.4	202.2	133.4	58.8	38.1	195.6	13.0	5.9	4.7	17.6
Histidine	31.4	14.3	12.4	67.5	39.6	19.6	10.8	69.3	2.6	1.6	0.5	4.4
Isoleucine	10.0	6.9	1.0	27.0	26.2	16.0	7.5	53.1	2.0	0.9	0.8	2.7
Leucine	30.0	12.3	12.7	60.6	59.8	33.1	18.9	114.8	2.3	1.3	0.8	3.8
Lysine	33.0	21.6	5.8	84.6	316.0	168.6	45.0	543.5	20.3	12.1	2.2	27.1
Methionine	6.9	5.4	0.8	18.1	81.4	25.0	42.5	118.4	<LLOQ	-	-	-
Phenylalanine	12.9	4.5	5.8	19.8	39.8	20.5	13.5	71.6	<LLOQ	-	-	-
Proline	40.4	15.8	17.6	82.5	424.4	108.5	221.7	588.7	12.8	1.3	10.9	13.9
Serine	97.6	28.8	55.4	143.6	204.2	67.5	73.9	291.1	3.2	2.0	0.8	5.4
Taurine	310.6	142.9	168.3	743.0	181.3	25.6	141.9	220.0	7.0	7.4	0.8	16.5
Threonine	81.5	27.7	45.4	132.0	309.3	122.4	99.3	528.4	3.5	2.0	0.8	5.7
Tryptophan	3.4	1.7	0.8	6.2	31.0	13.8	11.0	55.0	1.5	0.5	0.8	1.9
Tyrosine	15.6	11.0	0.8	35.3	67.4	32.8	21.4	119.8	<LLOQ	-	-	-
Valine	51.5	17.4	27.1	93.6	98.8	45.6	37.6	166.5	4.7	3.1	0.8	7.8
**Total (μM)**	**3,031.6**	**775.0**	**1,997.7**	**4,569.4**	**3,460.2**	**1,028.1**	**1,557.9**	**4,868.7**	**240.5**	**90.8**	**169.3**	**367.1**
EAA (μM)^b^	274.6	94.2	177.0	524.0	1 055.6	464.8	300.2	1 826.0	49.0	25.3	11.7	65.1
**EAA/Total (%)**	**9.1**	**2.2**	**6.3**	**12.8**	**29.2**	**5.8**	**19.3**	**37.5**	**21.2**	**13.1**	**6.5**	**38.0**
NEAA (μM)^b^	2,757.0	715.8	1,767.2	4,192.2	2,404.6	585.7	1,257.6	3,042.7	191.5	82.1	105.0	302.0
**NEAA/Total (%)**	**90.9**	**2.2**	**87.2**	**93.7**	**70.8**	**5.8**	**62.5**	**80.7**	**78.8**	**13.1**	**62.0**	**93.5**
**EAA/NEAA**	**0.10**	**0.03**	**0.07**	**0.15**	**0.42**	**0.11**	**0.24**	**0.60**	**0.30**	**0.23**	**0.07**	**0.61**
**Glx/Total (%)**	**60.6**	**5.5**	**46.4**	**69.3**	**12.2**	**1.5**	**10.2**	**13.9**	**55.6**	**15.4**	**34.8**	**71.8**
SAA (μM)^c^	335.0	143.8	181.9	757.6	295.8	60.5	222.8	378.1	8.5	7.4	2.3	18.0
**SAA/Total (%)**	**11.7**	**5.9**	**5.8**	**27.1**	**9.0**	**2.4**	**7.4**	**14.7**	**3.9**	**4.1**	**0.9**	**10.0**

^a^Glx: glutamine and glutamic acid.

^b^EAA: Essential Amino Acids (Sum of His, Arg, Ile, Leu, Lys, Met, Phe, Thr, Trp, Val concentration), NEAA: Non-Essential Amino Acids (Sum of Ala, Asp, Asn, Glu, Gln, Cys-Cys, Pro, Gly, Ser, Tyr, Cit, Tau concentration) , these EAA were considered as essential for human.

^c^SAA: Sulfur Amino Acids : taurine. cystine and methionin.

## Discussion

The present work describes the development, optimization, and validation of a micro assay for the quantitation of 21 free amino acids in human and mammalian milk using UPLC-ESI-MS/MS. Whereas FAA have been quantitated in human and cow milk with numerous analytical methods in earlier studies, the current report is first to provide data for rat milk free amino acids. In the current study, to ensure accurate quantitation of amino acids in biological samples, all validation steps were carried out in the milk samples, and the matrix effect was measured by the calculation of recovery for all FAA. The high recoveries (>95%) obtained in milk are similar to those achieved using other methods (Agostoni et al. 2000; Carratù et al. 2003). In addition, the limits of detection (LOD) in human milk (between 0.2 - 0.9 μmol/L) are significantly lower than those (3.3 μmol/L) reported by Carratù *et al*. ( 2003), and Agostoni *et al*. (2000) using HPLC-fluorescence detection. At the lower limit of quantitation (LLOQ), the precision was better than 10% except for cystine.

We observed striking interspecific differences between mature milks of human, ruminant, and rodent regarding both total FAA (from 240 to 3460 μM), the nature of the most abundant FAA, the EAA/NEAA ratio (from 10% to 42%), and the percentage of sulfur amino acids (from 4% to 11.7%).

The human milk samples obtained from our biocollection originated from lactating mothers donating their milk during the first two months of lactation. Taking into account the inter-individual variations, the FAA levels observed are very similar to those obtained in previous studies using a Beckman amino acid analyzer (Atkinson et al. 1980; Chuang et al. 2005) or HPLC-Fluorescence (Pamblanco et al. 1989), and higher than the average 1189 μM reported by Pamblanco et al. (1989) using HPLC-UV. The method used in our mammalian milk analysis yields results similar to those obtained with other methods such as reversed-phase HPLC, amino acid analyzer, and ion exchange chromatography amino acid analyzer. Consistent with previous studies (Atkinson et al. 1980; Chuang et al. 2005; Sarwar et al. 1998), we found Glx, taurine and alanine to be the three most abundant FAA, and the EAA/NEAA ratio between 11 and 13% concurs with published values as well (Carratù et al. 2003).

**Figure 4:**
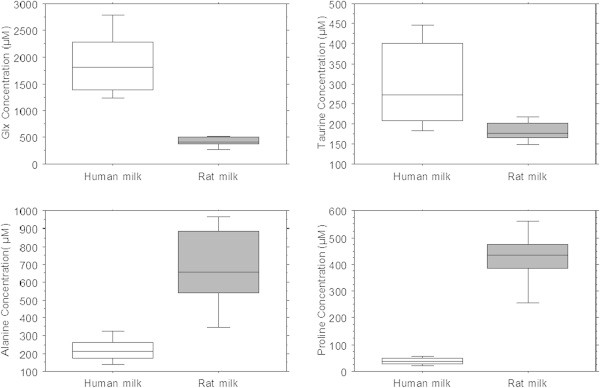
**Box and whisker plots showing the distribution of concentrations of main FAA (Glx, Alanine, Taurine and Proline) in human and rat milk.**

Although the composition of many mammalian milks has been analyzed (Sarwar et al. 1998), to the best of our knowledge, the current study is first to report the FAA concentrations of rat milk. Whereas Glx was the most abundant amino acid in human and bovine milk, alanine was the most abundant amino acid in rat milk, followed by proline and Glx (Figure 4). The higher EAA/NEAA ratio observed in rat milk (42%) is consistent with the view that rat pups are born more immature than human infants and calves, and therefore are more comparable to human premature neonates with higher nutritional requirements and faster growth rates.

In cow milk, we found lower total FAA (240 μM) than reported in the few earlier studies analyzing FAA in bovine milk (350 - 460 μM (Jensen 1995) to 1061 μM (Sarwar et al. 1998)). In addition, although two studies (Lindmark-Mansson et al. 2003; Sarwar et al. 1998) found Gln, and Tau to be nearly as abundant, we found Glx was by far the most abundant FAA, with much lower levels of other amino acids such as Ala, Gly, Lys, Pro, and Tau in bovine milk. The striking differences observed in free amino acid content and composition between bovine and human milk raise an important issue: does the specific free amino acid composition of human milk provide any health benefit? If so, should infant formulas, which are prepared from bovine milk, be supplemented with specific FAA (besides taurine)? These questions clearly warrant further investigation.

As a matter of fact, although milk FAA can be considered as micronutrients, several amino acids are suspected to have physiological effects in the infant, especially in the first few months after birth. A few animal experiments and clinical trials of FAA supplementation have confirmed these effects. This is particularly the case for glutamine and alanine, the major FAA present in the milk of the three species tested. Glutamine is the most abundant amino acid in the body accounting for about 60% of FAA in human body, and plays many documented roles. For instance, glutamine is a major fuel for the enterocyte, a substrate for purine and pyrimidine nucleotide synthesis, a regulator of protein synthesis (Hankard et al. 1996), and a major donor of carbon for gluconeogenesis (Hankard et al. 1997). Glutamine also supports the function of gut associated intestinal mucosal system (Wu and Knabe 1994), and gut redox state (Humbert et al. 2007), and a role of glutamate has recently been proposed for glutamate in the regulation of food intake (Ventura et al. 2012). Studies performed in human infants have shown that glutamine is extensively taken up by splanchnic tissues in the first few days of life even in preterm infants with a very low birth weight (Darmaun et al. 1997). Supplementation of infant enteral feeding with glutamine was shown to decrease infectious morbidity but not feeding tolerance in very-low birth weight infants (van den Berg et al. 2005). Alanine, a non essential amino acid in human, is a major substrate involved in gluconeogenesis, and in the citric acid cycle. In a rodent model, alanine was shown to compete for the taurine transport site and to reduce renal cortex taurine content after its ingestion (Chesney 1988). Taurine and lysine, the two other prominent FAA in maternal and bovine milk, respectively, are considered key nutrients for growth and development. Through its role in bile acid conjugation, taurine plays a key role in intestinal fat absorption. Taurine also plays a role in the protection of neurons against oxidative damage (Huxtable 1992; Wharton et al. 2004) although its beneficial effect on long-term neurodevelopmental outcome has still to be proven for preterm infants (Verner et al. 2007). The second most abundant FAA in milk rat, proline, is a glucogenic amino acid, a precursor of the polyamines, which play a key role in gut maturation in human neonates (Plaza-Zamora et al. 2013). In addition, proline is a precursor of arginine (Tomlinson et al. 2011), the sole endogenous precursor of nitric oxide, which plays a critical role in the regulation of intestinal blood flow. Arginine supplementation was suggested to decrease risk of necrotizing enterocolitis in preterm infants (Amin et al. 2002). Wu *et al* (2011) showed the nutritional importance of the arginine–proline cycle for the growth and development of piglet intestine, and supplementation in arginine increased sow’ milk production and enhanced the growth of suckling piglets (Mateo et al. 2008).

In conclusion, in this paper, we propose an innovative method to analyze FAA in mammalian milk. The fast sample preparation and short run time, and the low lower limit of detection are definite assets of the method. We are first to report the free amino acid composition of rat milk, and the ability to quantitate free amino acids in microsamples of milk is a definite advantage to assess milk composition in rodents. Regarding human milk, the monitoring of free amino acid composition may be relevant to the nutritional management of human infants. Many free amino acids indeed are thought to exert beneficial effects on infant physiology, but there is a wide range of variation between individuals; so ensuring specific free amino acids are present in a given maternal milk, at concentrations high enough to exert a physiological effect may prove helpful. Moreover, while many studies have shown that maternal diet can alter the fatty acid composition in human milk, very little is known about the potential manipulation of maternal protein and amino acid diet to alter milk amino acid composition, although a study of Guatemalan mothers suggests the source of protein (predominantly from plants vs. animal protein) dietary intake can modulate the free amino acid content of human milk (Wurtman and Fernstrom 1979). Exploring such relationship would clearly be warranted, in view of the increasing interest for research on maternal milk and its health benefits for human infants.
